# Decreased phenol sulfotransferase activities associated with hyperserotonemia in autism spectrum disorders

**DOI:** 10.1038/s41398-020-01125-5

**Published:** 2021-01-07

**Authors:** Cécile Pagan, Marion Benabou, Claire Leblond, Freddy Cliquet, Alexandre Mathieu, Nathalie Lemière, Hany Goubran-Botros, Richard Delorme, Marion Leboyer, Jacques Callebert, Thomas Bourgeron, Jean-Marie Launay

**Affiliations:** 1 Service de Biochimie et Biologie Moléculaire, INSERM U942, Hôpital Lariboisière, AP-HP, Paris, France; 2 Fondation Fondamental, Créteil, France; 3 Human Genetics and Cognitive Functions Unit, Institut Pasteur, UMR 3571, CNRS, Université de Paris, Ecole Doctorale Bio Sorbonne Paris Cité, Paris, 75015 France; 4 Université de Paris, Paris, France; 5 Child and Adolescent Psychiatry Department, Hôpital Robert Debré, AP-HP, Paris, France; 6 Psychiatry Department, Hôpital Henri Mondor-Albert Chenevier, AP-HP, Université Paris Est, Créteil, France; 7 INSERM U955, Psychiatrie Translationnelle, Université Paris-Est, Créteil, France; 8 Present Address: Service de Biochimie et Biologie Moléculaire, Centre de Biologie et de Pathologie Est, Hospices Civils de Lyon, 69500 Bron, France

**Keywords:** Autism spectrum disorders, Biomarkers, Physiology, Genetics

## Abstract

Hyperserotonemia is the most replicated biochemical abnormality associated with autism spectrum disorders (ASD). However, previous studies of serotonin synthesis, catabolism, and transport have not elucidated the mechanisms underlying this hyperserotonemia. Here we investigated serotonin sulfation by phenol sulfotransferases (PST) in blood samples from 97 individuals with ASD and their first-degree relatives (138 parents and 56 siblings), compared with 106 controls. We report a deficient activity of both PST isoforms (M and P) in platelets from individuals with ASD (35% and 78% of patients, respectively), confirmed in autoptic tissues (9 pineal gland samples from individuals with ASD—an important source of serotonin). Platelet PST-M deficiency was strongly associated with hyperserotonemia in individuals with ASD. We then explore genetic or pharmacologic modulation of PST activities in mice: variations of PST activities were associated with marked variations of blood serotonin, demonstrating the influence of the sulfation pathway on serotonemia. We also conducted in 1645 individuals an extensive study of *SULT1A* genes, encoding PST and mapping at highly polymorphic 16p11.2 locus, which did not reveal an association between copy number or single nucleotide variations and PST activity, blood serotonin or the risk of ASD. In contrast, our broader assessment of sulfation metabolism in ASD showed impairments of other sulfation-related markers, including inorganic sulfate, heparan-sulfate, and heparin sulfate-sulfotransferase. Our study proposes for the first time a compelling mechanism for hyperserotonemia, in a context of global impairment of sulfation metabolism in ASD.

## Introduction

**Fig. 1:**
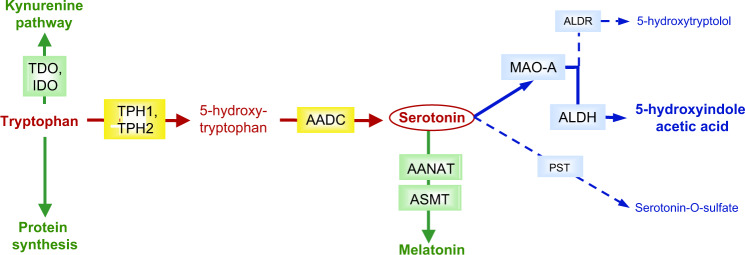
Serotonin metabolism. Serotonin is synthesized from tryptophan (red steps) by tryptophan hydroxylases (TPH1, non-neuronal, and TPH2, neuronal) and aromatic amino-acids decarboxylase (AADC). Of the two catabolic pathways (blue steps), the major one sequentially involves monoamine oxidase A (MAO-A) and aldehyde dehydrogenase (ALDH) or reductase (ALDR). Sulfoconjugation by phenol sulfotransferases (PST) is a quantitatively minor pathway. Alternatively, tryptophan can enter the kynurenine pathway after oxidation by indoleamine-2,3-dioxygenase (IDO) or tryptophan 2,3-dioxygenase (TDO), or serotonin be converted into melatonin by arylalkylamine N-acetyltransferase (AANAT) and acetylserotonin methyltransferase (ASMT).

According to the *Diagnostic and Statistical Manual of Mental Disorders*, fifth edition (DSM-5), the core symptoms of autism spectrum disorders (ASD) comprise deficits in social communication and interaction, and repetitive and restricted behaviors, which include sensory abnormalities. The heritability of ASD is high (>80%)^1^ and its genetic architecture is made of a combination of both rare and common variants^2^. The rare mutations mostly converge in specific biological pathways such as synaptic function or chromatin remodeling and modify synaptic plasticity and neuronal connectivity during brain development^3^. Although variants in hundreds of different genes have been reported, a genetic known cause is detected in less than 25% of individuals with ASD and in most of the cases the etiology remains unknown. In this highly heterogeneous context, the identification of recurrent biological features (i.e., biological endophenotypes) or recurrently affected pathways can be a key to understand the mechanisms underlying ASD. Among the biochemical endophenotypes described in ASD, hyperserotonemia^4–8^ is the most replicated^9,10^ and is reported in almost half of the patients^4–8^. However, the mechanism(s) of increased blood serotonin (5-hydroxytryptamine, 5-HT) in a subset of individuals with ASD remain(s) not clearly elucidated, although serotonin metabolism (Fig. 1) and transport have been widely investigated by biochemical and genetic approaches. At a genetic level, candidate gene approaches - including *SLC6A4*, encoding the serotonin transporter [SERT] or *MAOA*, encoding the A isoform of monoamine oxidase [MAO], the main serotonin catabolic enzyme - or high throughput approaches failed to explain this feature. Rare mutations and frequent polymorphisms with a functional impact were identified, but these variants could not be associated with modification of whole blood or platelet serotonin level^11–13^.

Besides the major MAO-mediated oxidative deamination, the second most important serotonin catabolism is sulfation^14,15^. For instance about 10% of serotonin in human blood platelets is sulfoconjugated^16^. Serotonin sulfation has not been studied in ASD, but several studies report alterations of sulfate metabolism with an increased urinary excretion of sulfate, sulfite and thiosulfate^17^, and a decreased sulfate content in the plasma^18^. An heparan sulfate (HS) deficiency has also been described in 4 post-mortem brain tissues of individuals with ASD^19^. Enzymatic sulfation of serotonin involves phenol sulfotransferases (PST), enzymes regulating the activities and terminal half-lives of thousands of metabolites, including neurotransmitters, hormones, and many exogenous compounds^20,21^. The PST-M or thermolabile (TL) isoform (encoded by the *SULT1A* genes, mainly *SULT1A3/SULT1A4*) is involved in the catabolism of bioamines (e.g., catecholamines or serotonin), whereas the PST-P or thermostable (TS) isoform (encoded by the *SULT1A* genes, mainly *SULT1A1* and *SULT1A2*) is involved in the catabolism of small phenolic chemicals^20,21^, though both variants will sulfate alternative substrates at high concentrations^22^. Here we investigated sulfation in blood samples from a large cohort of patients, their first degree relatives and control individuals from the general population, as well as in post-mortem tissues (from two major sources of serotonin, the gastro-intestinal tract, and the pineal gland) from individuals with ASD and controls. We showed that PST activities were decreased in patients with ASD, and that this decrease was correlated with hyperserotonemia, thus proposing a compelling mechanism for hyperserotonemia in ASD.

## Subjects and methods

### Subjects, clinical evaluations, blood, and autopsy-derived tissue samples

Characteristics of the autopsy-derived tissue samples (ilea and pineal glands) as well as clinical evaluations and blood sampling of individuals with ASD (diagnosed according to DSM-IVTR), their first degree relatives, and control individuals from the general population investigated for genetics and blood biochemistry have been detailed previously^8,23^. For the majority of the participants, the psychiatric and cognitive evaluations was performed the same week as the blood collection. For blood investigations, the difference in patients’ numbers between this study and previous ones^8,23^ are indicated in Supplementary Table 1. The ancestry of the participants for the genetic study is indicated in Supplementary Fig. 1).

The local Institutional Review Boards (Comité de Protection des Personnes Ile de France IX) approved this study. Written informed consents were obtained after oral and written information from all participants of the study, and from the children’s parents when subjects were under 18.

### Mice

Citrated blood from the mouse strains FVB/N [wild type], *Sult1a1* knockout [ko], and transgenic for human *SULT1A1/2* [tg]^24,25^ in this background as well as from 17α-ethinylestradiol(EE2)-treated tg mice (daily i.p. injection of 100 nM EE2 during 1 week)^26^ were from the German Institute of Human Nutrition. Mice were bred and underwent experiments respecting European guidelines for the care and ethical use of laboratory animals (Directive 2010/63/EU of the European Parliament and of the Council of 22 September 2010 on the protection of animals used for scientific purposes). Whole blood serotonin was measured in each blood sample (*n* = 5 mice for each strain).

### Biochemistry

PST (EC 2.8.2.1) activities were determined by radio-enzymology using either serotonin (PST-M) or p-nitrophenol (PST-P) as substrates^27^. PST-M activity was reported to reflect mostly SULT1A3 activity but, in fact, measures all SULT1A activities^28^. Platelet PST amounts were determined by ELISA (LS-F30288 and LS-F52687 from Life Span Biosciences for SULT1A1 [mainly PST-P] and SULT1A3 [mainly PST-M] respectively) according to manufacturer’s instructions. Plasma total inorganic sulfate levels were measured radiochemically^29^. Heparan sulfate (HS) contents were determined in plasma and autopsy-derived tissues by a sandwich enzyme immunoassay (Cat. no.280564-1, AMS Biotechnology, UK) using two monoclonal antibodies specific to HS and performed according to the manufacturer’s instructions.

Whole blood and tissue 5-HT and 5-hydroxyindole acetid acid (5-HIAA, the primary catabolite of 5-HT) were measured by high-performance liquid chromatography^30^. Plasma noradrenaline was measured by radioenzymology^31^. Tryptophan hydroxylase (TPH - EC 1.14.16.4), aromatic aminoacid decarboxylase (AADC - EC 4.1.1.28), MAO-A (EC 1.4.3.4) and heparin *O*-sulfotransferase (HST - EC 2.8.2.29/30) activities were measured in autopsy-derived tissues by radioenzymology. Analyses were performed blinded. Technical details of enzymatic assays are included as supplementary information.

### Genetics

The study of *SULT1A* genes (*SULT1A1*, *SULT1A2*, and *SULT1A3-4*), i.e. analysis of copy number variants (CNVs) and sequencing of coding exons 2 to 8 of *SULT1A1*, *SULT1A2* and *SULT1A3-4* as well as non-coding exons 1A, 1B and 1C of *SULT1A3*, is detailed in supplementary methods and Supplementary Tables 2 and 3.

### Statistical analyses

Statistical analyses were conducted using JMP Pro 11 software (SAS). Because most biochemical parameters are not normally distributed, non-parametric statistical tests were preferred. Two-sided tests were performed and error type I was chosen at 0.05. The statistical tests used for this study were Wilcoxon two-sample test, Kruskal-Wallis test, Pearson’s chi-square test, Fisher’s exact test, and linear regression.

## Results

### Deficient phenol sulfotransferase activities in patients with ASD

**Fig. 2:**
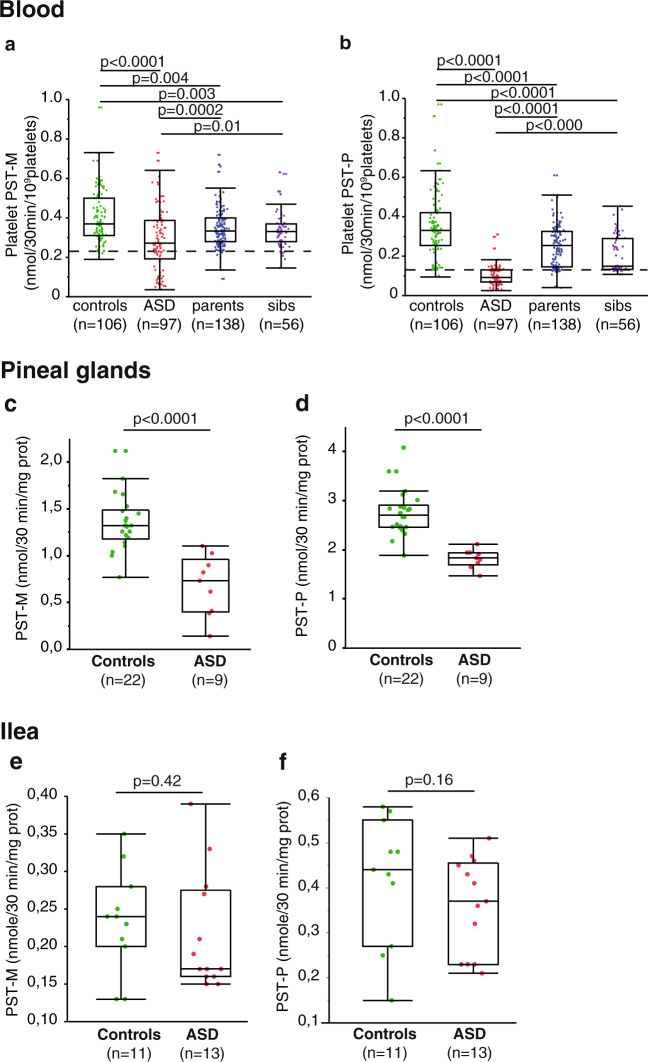
Phenol sulfotransferase activities in blood, post-mortem pineal glands, and ileal samples from patients with ASD. **a**–**c** Blood samples were taken in the morning from 97 unrelated individuals with ASD, their first-degree relatives (138 parents and 56 unaffected sibs), and 106 controls. **a** Platelet PST-M activities. **b** Platelet PST-P activities. **c**, **d** Pineal gland samples were obtained from 9 patients with ASD and 22 age- and sex-matched controls. **e**, **f** Ileal samples were obtained from 13 patients with ASD and 11 matched controls. Boxes indicate medians and quartiles. Dashed lines indicate the threshold of the 5th percentiles of the control group. Groups were compared using the Wilcoxon two-sample test.

Analysis of platelet PST activities was performed in 397 individuals, including 97 patients with ASD, 138 parents, 56 unaffected siblings and 106 sex- and age-matched controls. As compared with controls, both PST activities (M and P) were significantly decreased in individuals with ASD and also in their first degree relatives (Fig. 2a, b). A positive correlation was observed between PST-M activities of individuals with ASD and those of their fathers [*ρ* = 0.591, *p* < 0.01, *n* = 55] but not those of their mothers. Positive correlations were also observed between PST-P activities of individuals with ASD and their fathers [*ρ* = 0.377, *p* < 0.05, *n* = 55], mothers [*ρ* = 0.400, *p* < 0.05, *n* = 54] and unaffected siblings [*ρ* = 0.644, *p* < 0.01, *n* = 56]. Taking as a threshold the 5th percentile of the control group (0.23 nmol/30 min/10^9^ platelets) we observed a low PST-M activity in 35% of individuals with ASD (no difference between girls [*n* = 16] and boys [*n* = 81]), 4% of parents (no difference between mothers and fathers), and in 11% of the siblings (Fig. 2a). For PST-P activity, 78% of individuals with ASD (no difference between girls and boys), 7% of parents (no difference between mothers and fathers), and 27% of the siblings presented values below the 5th percentile control threshold (0.13 nmol/30 min/10^9^ platelets, Fig. 2b). Platelet PST amounts, determined in 32 samples, did not differ between individuals with ASD and controls (Supplementary Fig. 2a, b).

PST expression is quite ubiquitous and blood platelets are a quantitatively minor source of these enzymes. We investigated whether a PST decrease was also detected in major serotonin-producing tissues, focusing on the gastro-intestinal tract and pineal gland. PST-M and PST-P activities were also found significantly decreased in autopsy-derived pineal glands (Fig. 2c, d) from individuals with ASD as compared to controls. The absence of significant variation in ileal samples (Fig. 2e, f) may be due to either tissue-dependent differential regulations or a lack of PST stability in post-mortem intestinal tissues. Indeed, although PST are reported to be highly expressed in the gastro-intestinal tract^32^, the activities measured here are similar to those of blood platelets and much lower than in pineal glands.

### Contribution of platelet PST activities to the hyperserotonemia of patients with ASD

**Fig. 3:**
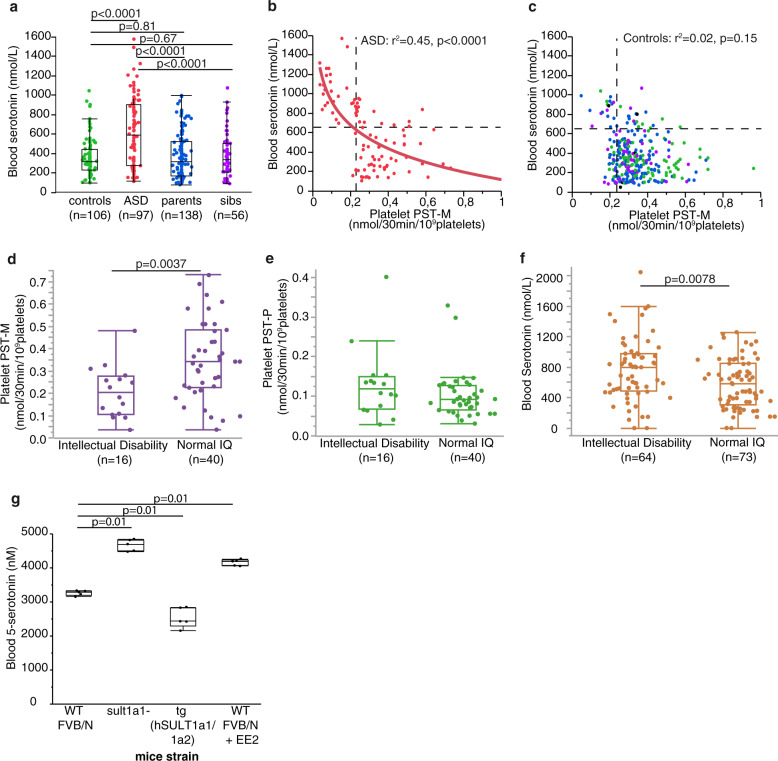
Relationship between PST activity and whole blood serotonin. **a** Whole blood serotonin. **b**, **c** Correlation between whole-blood serotonin concentration and platelet PST-M activity in **b** patients with ASD and **c** their relatives and controls (Pearson’s test after log transformation). **d**–**f** Platelet PST-M and PST-P activities and blood serotonin according to intellectual disability defined as verbal and performance IQ < 70. **g** Blood serotonin measured in wild type FVB/N mice (WT), in mice knockout for sult1a1 gene (sult1a1−), in mice transgenic for human SULT1A (tg hSULT1A1/1A2) and in wild-type FVB/N mice treated with 17α-ethinylestradiol (WT FVB/N + EE2), *n* = 5 in each group. Boxes indicate medians and quartiles. Groups were compared using the Wilcoxon two-sample test.

As compared to PST-M, platelet PST-P activity appeared more diminished in individuals with ASD compared to controls (Fig. 2a, b). However, platelet PST-M (but not PST-P) activity was strongly negatively correlated with the whole-blood serotonin level in individuals with ASD (Fig. 3a–c), suggesting that for these patients platelet PST-M activity might play a role in the regulation of whole-blood serotonin level. This negative correlation holds true for both girls (*ρ* = 0.653, *p* < 0.001, *n* = 16) and boys (*ρ* = 0.637, *p* < 0.001, *n* = 81) as well as for young (<16 y, *n* = 28, *ρ* = 0.389, *p* < 0.05) and older (>16 y, *n* = 69, *ρ* = 0.672, *p* < 0.001) individuals with ASD. In contrast, both PST-M and PST-P activities were not correlated with plasma noradrenaline (Supplementary Fig. 2c, d).

We then addressed if biochemical parameters were correlated to cognitive and clinical features. Interestingly, individuals with ASD and intellectual disability exhibited a lower platelet PST-M (but not PST-P) activity and a higher blood serotonin level than individuals with ASD and normal IQ (Fig. 3d–f, Supplementary Fig. 3). None of the biochemical parameters were significantly correlated to the Autism Diagnostic Interview-Revised (ADI-R) (Supplementary Fig. 4) or to the Repetitive Behavior Scale-Revised (RBS-R) (Supplementary Fig. 5) scores, but we could observe a negative correlation between blood serotonin level and the social responsiveness scales (SRS) scores (SRS total score *R*^2^ = −0.443, *p* = 0.049; Supplementary Fig. 6; of note a higher SRS score means higher severity).

We also investigated the activities of TPH, AADC, and MAO-A as well as the 5-HIAA content in post-mortem intestinal samples from patients and controls. No differences were observed between patients and controls for these parameters (Supplementary Fig. 7).

### Relationship between PST activities and serotonemia in mice

In mice, only the *Sult1a1* gene is present. In order to confirm the relationship between PST activities and serotonemia, we measured whole-blood serotonin in mouse strains either devoided of endogenous *Sult1a1* (ko) or carrying a transgene for the expression of human SULT1A1/1A2 (tg) and in tg mice treated by 17α-ethinylestradiol (EE2), a potent inhibitor of sulfotransferase 1A1. Indeed, the inhibition of sulfotransferase, either genetic or pharmacologic, resulted in increased whole-blood serotonin (Fig. 3g). This clearly asserts the influence of PST upon whole-blood serotonin level.

### Genetic studies of PST in humans

Considering that the biochemical impairment of PST activities observed in individuals with ASD is shared by first-degree relatives (Fig. 2a, b), we hypothesized that these impairments may be caused by genetic variants and hence performed a genetic analysis of PST genes. Interestingly, the *SULT1A* genes map at locus 16p11.2 (Supplementary Fig. 8), a chromosomal region with recurrent copy number variations associated with ASD: microdeletions and microduplications of this region are found in 1% of ASD patients, while their prevalence in the general population is less than 0.1%^22^. The deep characterization of the 16p genomic region is beyond the scope of our article, but we ascertained the genotype of the participants by quantifying the copy number of the SULT1A and by sequencing variants that could represent signatures of *SULT1A3* and *SULT1A4*. The duplication of the *SULT1A3* and *SULT1A4* is a recent event that appeared during primate evolution between the split of Chimpanzee and humans^33^. Therefore, both the exonic and intronic sequences are very similar.

Copy numbers of *SULT1A* genes (*SULT1A1*, *SULT1A2* and *SULT1A3-4*) were measured in a large cohort of 1,645 individuals, including 470 patients with ASD, 852 parents, 143 unaffected siblings, 45 affected siblings and 135 sex- and age-matched controls. We did not observe any association between *SULT1A* gene copy numbers and ASD status (Supplementary Fig. 9 and Supplementary Table 4). Coding regions of *SULT1A1*, *SULT1A2* and *SULT1A3-4* were sequenced in 264 individuals including 79 patients with ASD, 115 parents and 72 sex- and age-matched controls. Several non-synonymous variants already listed in the gnomAD (https://gnomad.broadinstitute.org/) were identified (rs1136703, rs10797300, rs9282861), but their frequencies were not significantly different between patients and controls. To test whether *SULT1A* CNVs influence PST activity, we compared the distributions of these traits between each *SULT1A* copy number in individuals with ASD and in controls. No significant difference could be observed between the groups (Supplementary Figs. 10 and 11). One female patient carried an homozygous deletion of *SULT1A1* and displayed normal PST-M activity (0.268 nmol/30 min/10^9^ platelet), but low PST-P activity 0.071 nmol/30 min/10^9^ platelet), in accordance with previous studies showing that the PST-P activity is a combination of the enzymes encoded by *SULT1A1* and *SULT1A2*^20,21^. In summary, the genetic analyses of *SULT1A* using sequencing and copy-number measure could not explain the reduction of PST activities observed in patients with ASD.

### Extended impairments of sulfation metabolism in ASD

**Fig. 4:**
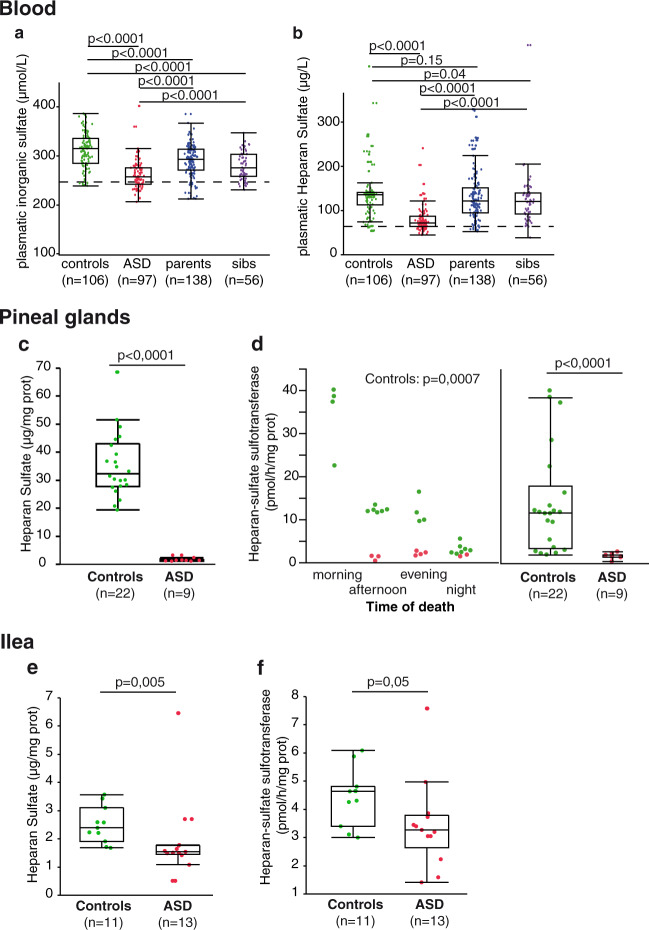
Sulfation metabolism in ASD. **a** Plasma inorganic sulfate concentrations. **b** Plasma HS concentrations. **c** Pineal HS contents. **d** Pineal heparan sulfate sulfotransferase activities, stratified by time of death (left panel) and pooled for comparison (right panel). **e** Ileal heparan sulfate contents. **f** Ileal heparan sulfate sulfotransferase activities. Boxes indicate medians and quartiles. Groups were compared using the Wilcoxon two-sample test.

In the absence of an obvious genetic explanation to the decrease of PST activities, we hypothesized a more general impairment of sulfation metabolism in ASD. Plasma inorganic sulfate (Fig. 4a) and heparan sulfate (HS) (Fig. 4b) levels were also decreased in individuals with ASD (31 and 28%, respectively under the control threshold values: 246 µg/L and 66 ng/mL with no difference between girls and boys), their parents (13 and 7% with no difference between mothers and fathers), and their siblings (14 and 5%). Whereas there is neither age nor gender difference for plasma sulfate^34^, plasma HS was inversely correlated with age until 25 years of age, without age-dependent changes thereafter^35^ and the most important decrease (54% as compared to the same age controls) was observed for individuals with ASD between 11 and 20 years. These parameters were not correlated with blood serotonin—indeed, the majority (59%) of patients with hyperserotonemia had normal plasma inorganic sufate and HS. Interestingly, when considering the four sulfation-related parameters (plasma inorganic sulfate and plasma HS concentrations, platelet PST-M and -P activities) as a whole, 85% of controls fit within the 95^th^ percentiles of the control group for all 4 parameters vs. only 9% of individuals with ASD. Biochemical profiles were highly heterogeneous among patients with ASD (Supplementary Table 5), suggesting complex mechanisms at the origin of these biochemical abnormalities.

As PST-M and PST-P activities (Fig. 2c, d), HS contents (Fig. 4c) but also heparin *O*-sulfotransferase (HST) activities (Fig. 4d) were found significantly decreased in autopsy-derived pineal glands from individuals with ASD as compared to controls. Of note control pineal HST activity showed a strong circadian regulation (Fig. 4d), inverse to that of melatonin synthesis as expected^36^. In autopsy-derived ilea, HS contents (Fig. 4e) and HST activities (Fig. 4f) were also found significantly decreased for individuals with ASD as compared to controls.

## Discussion

The present study reasserts that individuals with ASD present a low plasma inorganic sulfate content^18^. Previous studies showed that a vitamin/mineral supplement^37^ or Epsom salts (magnesium sulfate)^38^ were able to improve, but not normalize, free and total plasma sulfate in children with ASD. However, whereas in the rat 3′-phosphoadenosine-5′-phosphosulfate (PAPS) and sulfate availability limit sulfation capacity, in mice and humans sulfotransferase activity limits the maximum rate of sulfoconjugation^39^. In addition, we reported, for the first time to our knowledge, that a subset of individuals with ASD presented, for the three investigated tissues (plasma, pineal, and ileum), reduced HS contents (Fig. 4b, c, e) as well as decreased HST activities (Fig. 4d, f). This finding is in line with the reported increased excretion of glycosaminoglycans (including HS) in the urine of patients with ASD^40^ and with data from mouse models of autism suggesting a possible connection between autism and HS. For instance the level of HS immunoreactivity was found reduced in the brain tissue of BTBR T+tf/J mice^41^, a naturally occurring inbred strain exhibiting behaviors recapitulating the major symptoms of autism^42,43^. Another mouse model results from the elimination of HS from postnatal neurons by conditionally inactivating *EXT1*, the gene encoding an enzyme essential for HS synthesis found associated with ASD in a GWAS meta-analysis^44^ but not confirmed in a more recent GWAS^45^: HS was reported to be critical for normal functioning of AMPA glutamatergic synapses and its deficiency to mediate socio-communicative deficits and stereotypies characteristic for autism^46^. *NDST1*, another gene important for HS biosynthesis, and its Drosophila ortholog *sulfateless* were found associated with intellectual disability^47^ and ASD-like behaviors^48^. Moreover, a genetic association has been found between autism and the *HS3ST5* gene encoding one of the HS 3-O sulfotransferases in two cohorts of European ancestry^49^, and a genome-wide scan in 996 cases with autism identified four independent CNVs in the *GPC5/GPC6* gene cluster^50^, which encodes the glypicans 5 and 6, two members of a family of glycosylphosphatidylinositol-anchored HS proteoglycans. The sulfate moieties found on the HS chains of proteoglycans form specific patterns which regulate various aspects of cell growth, differentiation, adhesion, and migration by modulating interactions with diverse bioactive molecules, such as growth factors, morphogens, and cell-surface receptors, see ref. ^51^ for review. HS proteoglycans are expressed throughout brain development and play important roles in axon guidance, synaptic development, and function^52^. This is reminiscent of the genes involved in ASD which converge on common pathways altering synaptic homeostasis^53^. As a whole, our present findings about HS and its sulfation substantiate the suspected implication of this glycan in ASD^54,55^. Of note all glycosaminoglycans except hyaluronic acid are sulfonated. Thus, besides HS and HS proteoglycans, the sulfation deficit observed in individuals with ASD might affect other glycosaminoglycans and their corresponding proteoglycans resulting in large modifications of the extracellular matrix.

As we previously proposed^5^, the origin(s) of hyperserotonemia in autism appear(s) to be of metabolic origin, i.e., a decreased catabolism and/or an increased biosynthesis of serotonin. An increased 5-HT synthesis has never been evidenced in ASD patients, see refs. ^56,57^ for review. The main 5-HT catabolic pathway, leading to 5-HIAA, is oxidative deamination through MAO-A. Mice ko for MAO-A exhibited autistic-like behaviors which could be prevented by reducing serotonin levels at an early developmental age (P1–P7), see ref. ^58^ for review. In post-mortem cerebella and frontal cortexes of ASD patients MAO-A activity was significantly reduced only in 5 cerebella out of 18^59^. Such a deficit was not reported, by measuring 5-HIAA, at the blood or urine levels^5^. Accordingly, taking as a threshold the 5th percentile of the control group (9.9 nM), only 5 out of 67 ASD patients (7.5%) of the present cohort had low plasma 5-HIAA levels and no differences were observed between patients and controls for MAO-A activity as well as for 5-HIAA content in post-mortem intestinal samples. The present results clearly identify a sulfation deficit (decreased platelet PST-M activity) as a major contributor to hyperserotonemia, either in individuals with ASD (Fig. 3a) or in mice after genetic or pharmacological manipulation of PST activities (Fig. 3c). As expected^60^, this sulfation deficit cannot however account for the noradrenaline level found increased in the plasma of ASD patients^61,62^. The early expression of PST in different developing fetus tissues and their spatial and temporal expression patterns also suggest that they might play a role in many biological processes, including brain development^63,64^. However, neither CNVs, nor sequence variants of *SULT1A* genes could be significantly associated with ASD status or PST activity. Regarding *SULT1A* copy-number, previous studies identified frequent duplications of *SULT1A1* and the copy-number was positively correlated with the enzyme activity in platelets and kidney^65,66^ and with estrogen metabolism^67^. In contrast, the correlation between *SULT1A3-4* copy-number and its activity had never been investigated and our results failed to find such a correlation. Given the fact that each individual investigated carried at least three copies, we could expect that additional copies of the genes could moderately influence the quantity of active enzyme. These results thus exclude the hypothesis that a recurrent deletion of *SULT1A3* could cause the frequent PST activity deficit observed. More research is thus needed to determine the cause(s) of the low PST activities reported in the present study.

A few studies already reported a sulfation deficit in autistic children, mainly in vivo through the metabolism of paracetamol^68^. The authors stated that the PST enzyme itself does not appear to be lacking or genetically weakened (our present genetic findings are in complete agreement with this statement), but that it is lacking a sufficient supply of sulfate to attach the phenolic molecules. This cannot account for the low PST activities reported here since an excess of PAPS - the active form of sulfate - is added to our reaction mixtures. Similarly, the reported effects of age [the highest PST activities (M>P) are for the human fetus^69^], sex [higher PST-M activities for women^70^, higher PST-P activities for men^71,72^] and ancestry [higher PST-P activities for African Americans^73,74^] are unlikely to be confounding factors of our findings as the correlation between PST-M activity and blood serotonin was observed in all age and gender groups and our cohort is mostly from European descent. Most of cytosolic SULTs exist as dimers, either homo- or hetero-dimers^75^, and both SULT1A1 and SULT1A3 harbor allosteric sites allowing their inhibition by non-steroidal anti-inflammatory drugs^76,77^. This allows various possibilities for disturbed regulations leading to low PST activities which can affect either endogenous metabolites (for instance the decrease of urinary 6-sulfatoxymelatonin repeatedly found in ASD patients^8,78,79^ might well result from both a low melatonin production^8,23^ and a decrease of 6-hydroxymelatonin sulfation by PSTs^28,80^) or numerous environmental chemicals, the majority of which are PST substrates^81^. Interestingly these xenobiotics are considered as risk factors for autism^82,83^. Theoretically, it would be interesting to know whether PST activators, such as some phenolic acids^84^, might be beneficial for subjects with low PST activities.

Besides the presently reported decreased PST activities, blood hyperserotonemia was also reported to result from increased SERT activity due: (i) to the rare Ala56 ASD-associated gain-of-function mutation in the *SLC6A4* gene found in some ASD patients^13^ which altered the communication and social domains in knock-in mice^85^ or (ii) to a variant in the promoter of the *ITGB3* gene^86^ which encodes the β-chain of the platelet integrin α_IIb_β_3_ known to interact physically with SERT to promote its externalization and transport activity^87^. However, both brain hyperserotonemia (as in *Slc6a4* knockout mice)^88^ and hyposerotonemia (as in *Tph2* knockout mice)^89^ can induce ASD-relevant repetitive behaviors and socio-communication deficits. As previously stated^57^, this illustrates that bidirectional (enhancement or depletion) perturbations of serotonin availability during development could underlie the persistent behavioral ASD characteristics. Accordingly, 5-HT was recently shown to mediate permissive gene expression through histone serotonylation^90^ and 8 ASD-derived induced pluripotent stem cells exhibited altered chromatin accessibility^91^.

This study proposes for the first time a biochemical mechanism for hyperserotonemia in ASD, associated with decreased PST activities in a context of general impairment of sulfation metabolism. Many consequences can be expected from alterations of such a pleiotropic metabolism. Further assessments of other PST substrates in various tissues are needed to better evaluate the impacts of these findings in ASD.

## Supplementary information

Supplemental material
