# Supplementary material for: Decreased phenol sulfotransferase activities associated with hyperserotonemia in autism spectrum disorders
Source: Transl Psychiatry. 2021 Jan 7;11:23. doi: 10.1038/s41398-020-01125-5 (PMC7791095; doi:10.1038/s41398-020-01125-5)
Supplement: Supplementary file 1 — Supplemental material [file 41398_2020_1125_MOESM1_ESM.docx]

**Supplementary Information**

**Genetic and biochemical analysis of the phenol sulfotransferase enzyme in Autism Spectrum Disorders**

Cécile Pagan^1,2,3,4,5,^*, Marion Benabou^3,^*, Claire Leblond^3^, Freddy Cliquet^3^, Alexandre Mathieu^3^, Nathalie Lemière^3^, Hany Goubran-Botros^3^, Richard Delorme^2,3,6^, Marion Leboyer^2,7,8^, Jacques Callebert^1,4^, Thomas Bourgeron^2,3 #^, Jean-Marie Launay^1,2,4#^

**Methods of biochemical analyses**

**Methods of *SULT1A* genes analyses**

**Supplementary Figure 1:** Ancestry analysis of the participant of the study.

**Supplementary Figure 2:** PST proteins; correlation between plasmatic noradrenaline and platelet PST-activities in patients with ASD and controls.

**Supplementary Figure 3:** Correlations between verbal IQ (VIQ), performance IQ (PIQ), full scale IQ (FSIQ) and blood serotonin level, plasma heparan sulfate and inorganic sulfate levels, and platelet PST-P and PST-M activities for individuals with ASD.

**Supplementary Figure 4:** Correlations between subscale scores of the ADI-R algorithm and blood serotonin level, plasma heparan sulfate and inorganic sulfate levels, and platelet PST-P and PST-M activities in ASD individuals.

**Supplementary Figure 5:** Correlations between subscale scores of the Repetitive Behavior Scale Revised and blood serotonin level, plasma heparan sulfate and inorganic sulfate levels, and platelet PST-P and PST-M activities in ASD individuals.

**Supplementary Figure 6:** Correlations between subscale scores of the Social Responsiveness Scale (version 2) and blood serotonin level, plasma heparan sulfate and inorganic sulfate levels, and platelet PST-P and PST-M activities in ASD individuals.

**Supplementary Figure 7:** Serotonin synthesis and catabolism in post-mortem ilea samples.

**Supplementary Figure 8:** Phylogeny of the SULT1A/B/C gene family and location of the SULT1A1-4 genes in the human genome.

**Supplementary Figure 9:** *SULT1A1* and *SULT1A3-4* copy number distributions in patients with ASD, their relatives and controls from all ancestries.

**Supplementary Figure 10:** Comparison of PST-M and PST-P activities, blood serotonin, plasma heparan sulfate and plasma inorganic sulfate levels between *SULT1A1* and *SULT1A3-4* copy numbers in patients with ASD.

**Supplementary Figure 11:** Comparison of PST-M and PST-P activities, blood serotonin, plasma heparan sulfate and plasma inorganic sulfate levels between *SULT1A1* and *SULT1A3-4* copy numbers in the control group.

**Supplementary Table 1:** Description of the populations investigated for biochemical parameters in blood.

**Supplementary Table 2:** Primers, probes and enzymes used for CNVs detection of *SULT1A1-4* genes using droplet digital PCR.

**Supplementary Table 3:** Primers used for amplification and sequencing of *SULT1A1-4* genes.

**Supplementary Table 4:** *SULT1A1* and *SULT1A3-4* copy number distributions in patients with ASD, their relatives and controls from European ancestry.

**Supplementary Table 5:** Biochemical profiles observed in patients with ASD and controls.

**Methods of biochemical analyses**

Platelet or tissue lysis was achieved by by -SH-activated toxin treatment[^1^](#_ENREF_1). 150 hemolytic units of alveolysin purified to homogeneity (equivalent to about 375 ng or 6 pmoles of protein) were added (10:1) to platelet suspensions or tissue homogenates at 4°C and the suspensions warmed to 37°C for enzyme measurement. This amount of toxin binds to platelets or cell fragments at 4°C and elicits complete lysis at 37°C, since about 16 molecules of toxin are sufficient to lyse one human platelet[^2^](#_ENREF_2). Separate experiments showed that the addition of equivalent amounts of toxin to cells already disrupted by homogenization produced no change in the rates of enzyme activities measured at least in duplicate. Under the conditions employed here, enzyme rates were linear with time and with platelet or cell protein concentrations ranging from 0.01 to 0.1 mg of protein per mL.

MAO-A enzymatic activity was determined as previously reported for human fibroblasts[^3^](#_ENREF_3) by a radioenzymatic assay using [^14^C]-5-HT creatinine sulfate (1.96 GBq/mmol,Amersham GE Healthcare, Little Chalfont, UK, final concentration 20 µM) as substrate according to[^4^](#_ENREF_4).

TPH activity was measured using a radioenzymatic assay[^5^](#_ENREF_5) validated for croaker hypothalamic tissues. Tissue homogenate (25 μL) was added to a reaction mixture containing 0.05 mM tryptophan, 50 mM Hepes (pH 7.60), 5 mM DTT, 0.01 mM Fe(NH4)2(SO4)2, 0.5 mM 6-MPH4, 0.1 mg/ml catalase, and 3H-tryptophan (1 μCi/reaction). The enzymatic reaction was allowed to proceed at 37°C for 20 min. Unreacted tryptophan and the product 5-HTP were adsorbed with 500 μl of 7.5% charcoal in 1 M HCl at the end of the incubation. The samples were thoroughly vortexed and centrifuged at 14,000 × g for 2 min. The supernatant (350 μL) was centrifuged, and a 200-μl aliquot of the final supernatant was added to 3 mL of scintillation fluid and the radioactivity was measured by a liquid scintillation counter (Beckman LS 6000SC). Blank values were obtained by performing the reaction in the absence of tissue homogenate and in the presence of boiled homogenate. The counts per minute were converted to pmoles of 5-HTP formed per milligram of tissue per hour using the formula described by[^5^](#_ENREF_5).

AADC activity was quantified by measuring the conversion of tritiated 5-HTP to tritiated serotonin[^6^](#_ENREF_6). Each 100 µL reaction was carried out in a borosilicate culture tube (12 x 75 mm) that contained the followings: 15 µmoles of potassium phosphate, pH 7.80; 0.12 µmoles of EDTA; 0.025% BSA; and 1 nmole of prepurified L-5-hydroxy-[G-3H]-tryptophan. The decarboxylase reaction was initiated by the addition of 25 µL of platelet or tissue homogenate and then incubated for 15 min at 37°C. The blank tubes received 25 µL of BSA instead of platelet or tissue homogenate. The reaction was terminated by the addition of 75 µL of 2.5M potassium borate, pH 11.00 and 1.25 mL of toluene/isopentyl alcohol (3:1). After vortexing and centrifugation at 1,500g for 5 min, a 1 mL aliquot of the organic phase was transferred to another culture tube containing 0.25 mL of 2M formic acid. The tubes were vortexed, centrifuged, and the organic phases removed by aspiration. A 150 µL aliquot of the aqueous phase was then transferred to a scintillation vial containing 1 mL of 1M potassium phosphate, pH 7.10 and 10 mL of scintillation fluid. The vials were shaken and then quantified as a two-phase system by liquid scintillation spectrometry.

Heparin *O*-sulfotransferase (OST) activity. The heparin 3-OST activity was measured by LC-MS according to[^7^](#_ENREF_7).

**Methods of genetic analyses**

*SULT1A1-4* copy number variants (CNVs) analysis. *SULT1A* genes (*SULT1A1*, *SULT1A2* and *SULT1A3-4*) copy number (CN) were measured in a large cohort of 1,645 individuals including 470 patients with ASD, 852 parents, 143 unaffected siblings, 45 affected siblings and 135 sex- and age-matched controls using droplet digital PCR (ddPCR). We used the hydroxymethylbilane synthase gene (*HMBS*) as a reference for copy number variants. To our knowledge, no deletion or duplication has been reported for this gene in databases. DNA samples were first digested. Primers and TaqMan® probes are described in Supplementary Table 2. CN were analyzed using QuantaSoft (Biorad) software. In order to obtain natural numbers for each copy number, results were rounded to the nearest natural number N when they ranged between N ± 0.3. When the result did not range between these values, CN were considered as ambiguous and were not included in the analyses. One control individual presented a distal duplication of the 16p11.2 region. This duplication was confirmed using SNP array (Illumina, HumanOmni5Exome) CNV detection, which showed a 1Mb duplication including all *SULT1A* genes. This individual was subsequently removed from the analyses.

Sequencing *SULT1A1-4* coding regions. Coding exons 2 to 8 of *SULT1A1*, *SULT1A2* and *SULT1A3-4* as well as non-coding exons 1A, 1B and 1C of *SULT1A3* were sequenced in 264 individuals including 77 patients with ASD, 115 parents and 72 controls sex- and age-matched controls. Primers used for Sanger sequencing are shown in Supplementary Table 3. Twenty ng of DNA were amplified using Fastart Taq DNA polymerase (Roche). Sequencing Chromatograms were analyzed semi-quantitatively using Sequencher software (Gene Codes). A score from 0 to 4 was attributed to each variant, representing the increasing proportion of the alternative allele.

**
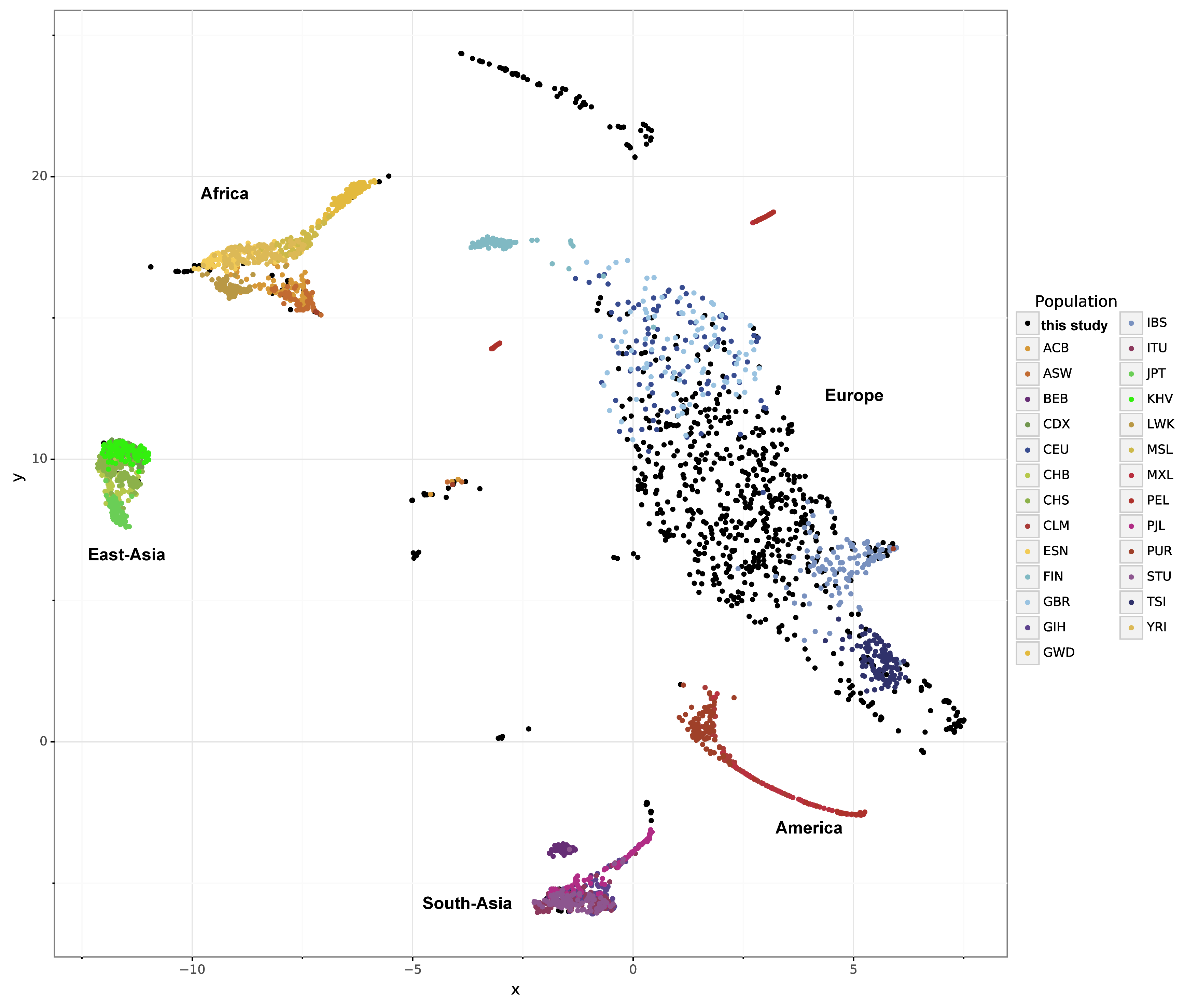
**

**Supplementary Figure 1: Ancestry analysis of the participant of the study.** Principal Components Analysis (PCA) of variance standardized relationship matrix was used to evaluate the ancestry of individuals in this study and to provide components for any covariate adjustments. To go further and cluster individuals based on ancestry, we further reduced dimensionality with uniform manifold approximation and projection (*UMAP; McInnes,L. et al. (2018) UMAP: Uniform Manifold Approximation and Projection for Dimension Reduction. arXiv:1802.03426*) reducing the first 8 PCA components to 2 components (x-umap-spread and y- umap-spread) that allows for better visualization and easier interpretation. Finally, to find subpopulation clusters (hdbscan column), we performed density based clustering (*HDBSCAN; Campello,R.J.G.B. et al. (2013) Density-Based Clustering Based on Hierarchical Density Estimates. In, Pei,J. et al. (eds), Advances in Knowledge Discovery and Data Mining, Lecture Notes in Computer Science. Springer Berlin Heidelberg, pp. 160–172.*). 1000 Genomes project studied 26 different populations from many different locations around the globe (<http://www.internationalgenome.org/category/population/>). These populations have been divided in five super populations: (1) African (AFR) including Yoruba in Ibadan – Nigeria (YRI, n = 108), Luhya in Webuye – Kenya (LWK, n = 99), Gambian in Western Divisions in the Gambia (GWD, n = 113), Mende in Sierra Leone (MSL, n = 85), Esan in Nigeria (ESN, n = 99), Americans of African Ancestry in SW USA (ASW, n = 61), African Caribbeans in Barbados (ACB, n = 96); (2) Ad Mixed American (AMR) including Mexican Ancestry from Los Angeles USA (MXL, n = 64), Puerto Ricans from Puerto Rico (PUR, n = 104), Colombians from Medellin – Colombia (CLM, n = 94), Peruvians from Lima – Peru (PEL, n = 85); (3) East Asian (EAS) including Han Chinese in Beijing – China (CHB, n = 103), Japanese in Tokyo – Japan (JPT, n = 104), Southern Han Chinese (CHS, n = 105), Chinese Dai in Xishuangbanna – China (CDX, n = 93), Kinh in Ho Chi Minh City – Vietnam (KHV, n = 99); (4) European (EUR) including Utah Residents (CEPH) with Northern and Western European Ancestry (CEU, n = 99), Toscani in Italia (TSI, n = 107), Finnish in Finland (FIN, n = 99), British in England and Scotland (GBR, n = 91), Iberian Population in Spain (IBS, n = 107); (5) South Asian (SAS) including Gujarati Indian from Houston – Texas (GIH, n = 103), Punjabi from Lahore – Pakistan (PJL, n = 96), Bengali from Bangladesh (BEB, n = 86), Sri Lankan Tamil from the UK (STU, n = 102), Indian Telugu from the UK (ITU, n = 102). The patients and the controls of this study are in majority from European descent (80.8% of the patients and 85.4% of the controls).


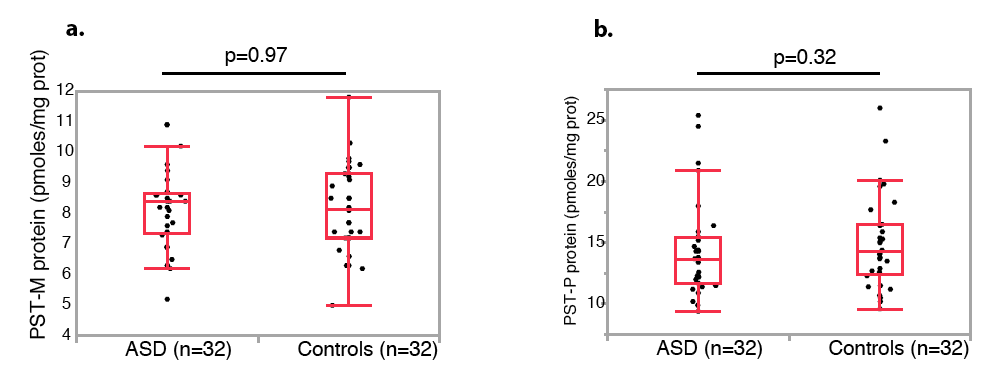


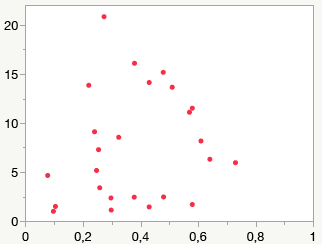

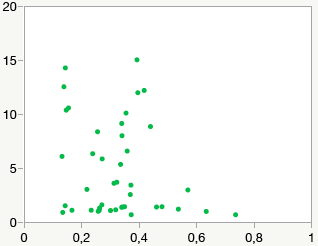

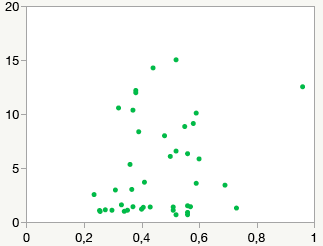


PST-M activity in platelets

(nmol/30 min/10^9^ platelets)

PST-P activity in platelets

(nmol/30 min/10^9^ platelets)

Plasma noradrenaline (nmol/L)

PST-M activity in platelets

(nmol/30 min/10^9^ platelets)

Plasma noradrenaline (nmol/L)

PST-P activity in platelets

(nmol/30 min/10^9^ platelets)

**c. Controls** (n=42)

r^2^=0.05, p=0.14

r^2^=0.05, p=0.17

Plasma noradrenaline (nmol/L)

Plasma noradrenaline (nmol/L)

**d. Patients with ASD** (n=26)

r^2^=0.05, p=0.28

r^2^=0.004, p=0.74


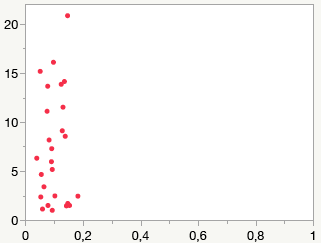


**Supplementary Figure 2: Platelet PST proteins (a,b); correlations between plasma noradrenaline and platelet PST-activities in controls (c) and patients with ASD (d).**


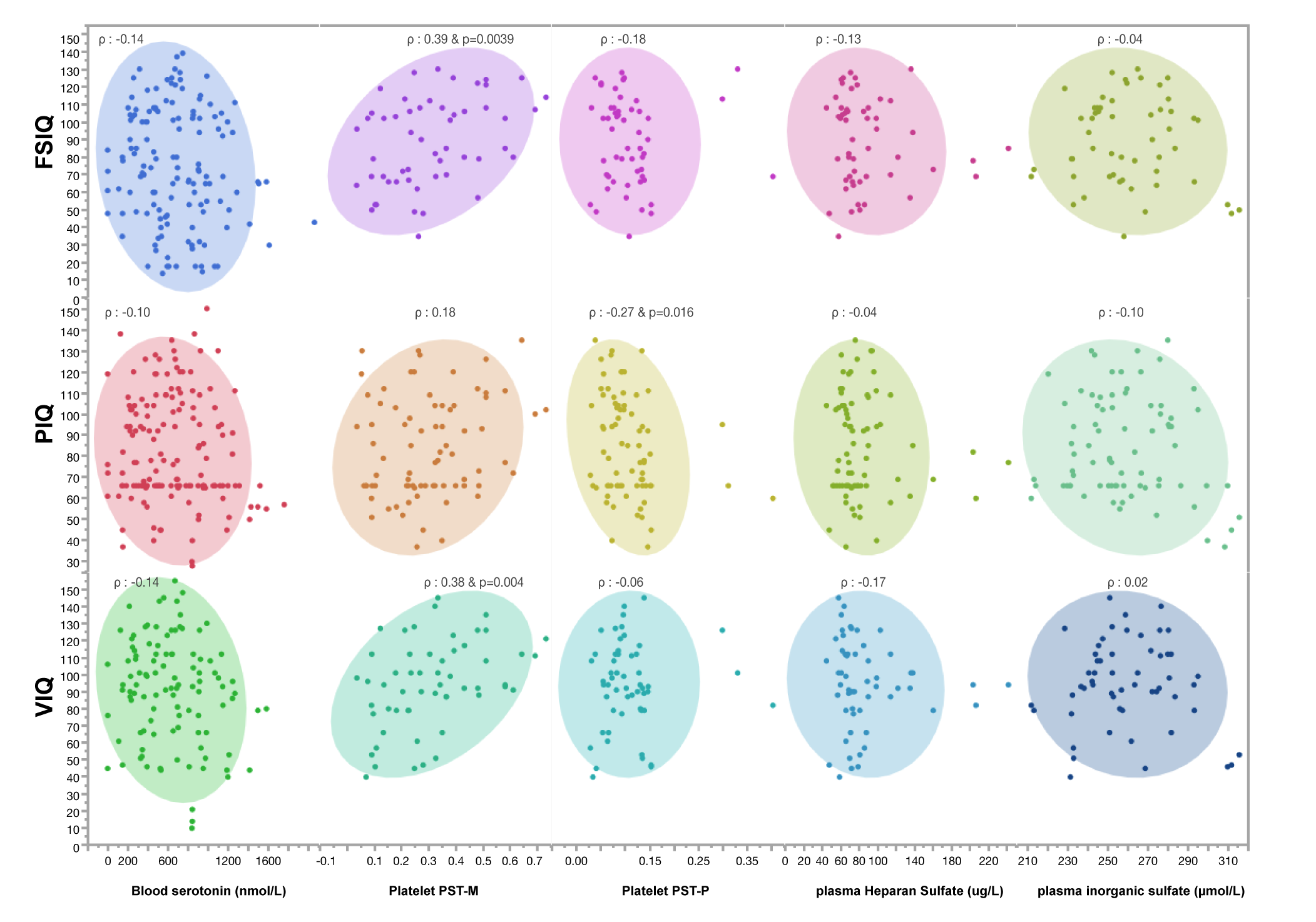


**Supplementary Figure 3: Correlations between verbal IQ (VIQ), performance IQ (PIQ), full scale IQ (FSIQ) and blood serotonin level, plasma heparan sulfate and inorganic sulfate levels, and platelet PST-P and PST-M activities for individuals with ASD.** IQ data were collected and blood serotonin measured on 151 individuals with ASD. Plasma heparan sulfate and inorganic sulfate levels, and platelet PST-P and PST-M activities were assessed within 77 individuals with ASD and IQ data. For each association, the Spearman's rank-order correlation coefficient (ρ) is indicated as well as significant nominal p values (p<0.05).

**
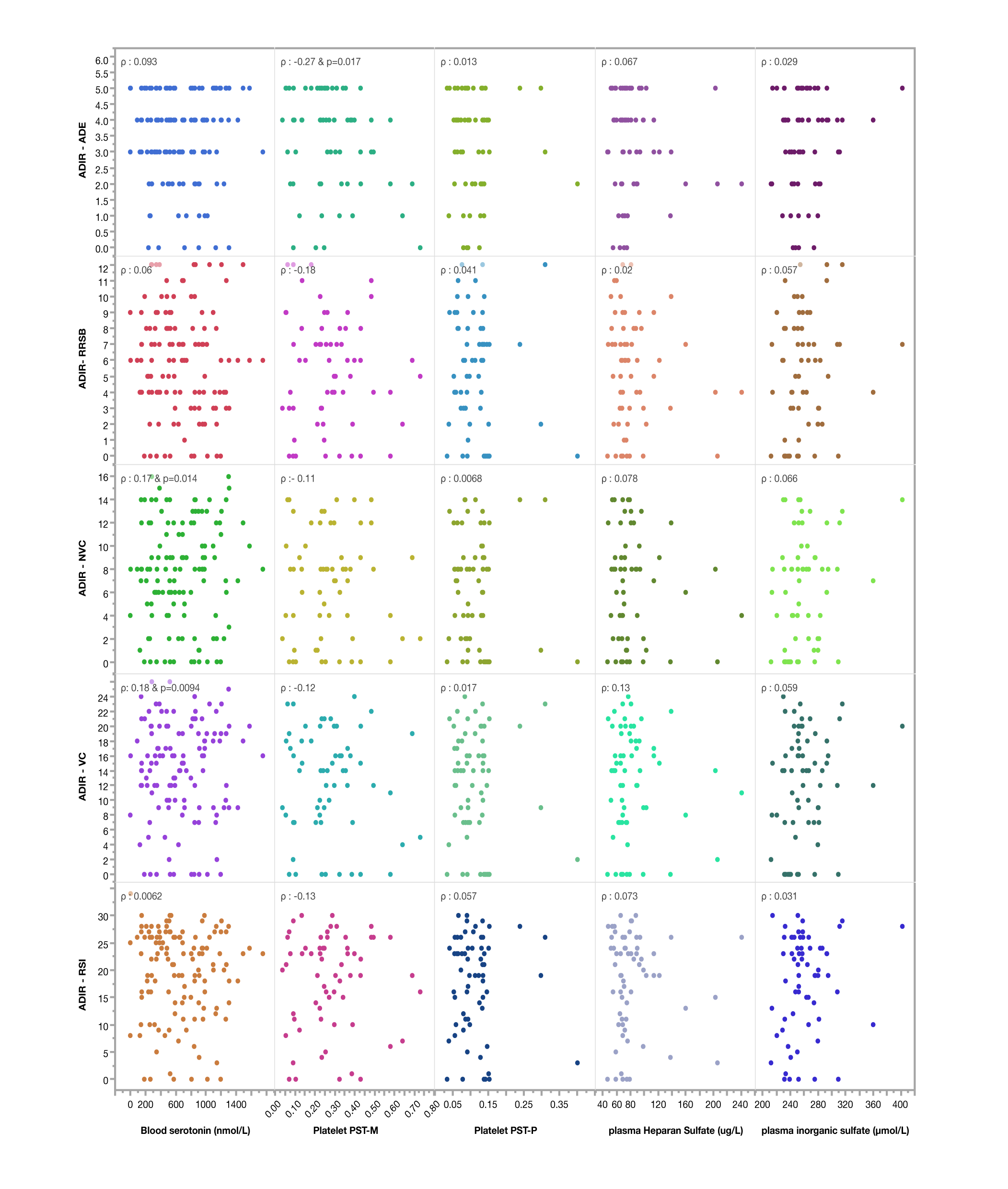
**

**Supplementary Figure 4: Correlations between subscale scores of the ADI-R algorithm and blood serotonin level, plasma heparan sulfate and inorganic sulfate levels, and platelet PST-P and PST-M activities in ASD individuals.** Blood serotonin was measured on 131 individuals with ASD. Plasma heparan sulfate and inorganic sulfate levels, and Platelet PST-P and PST-M activities were assessed within 65 individuals with ASD. The ADI-Scores are from infancy (ever). We used the non-parametric Spearman's rank-order correlation to measures the strength and direction of association between the ADI-R items and the biochemical variables. Correlations should however be taken with great care since most of the ADI-R items are not continuous variables and a linear relationship between the ADI-R items and the biochemical variables might not exist. ADI-R, Autism Diagnostic Interview-Revised; RSI, Reciprocal Social Interaction; VC, Verbal Communication; NVC, Non-Verbal Communication; RRSB, Restricted, Repetitive, and Stereotyped Patterns of Behavior; ADE, Abnormality of Development Evident at or before 36 months. For each association, the Spearman's rank-order correlation coefficient (ρ) is indicated as well as significant nominal p values (p<0.05). After correction for multiple testing, none of the biochemical parameters are significantly associated with the ADI-R subscale scores.

**
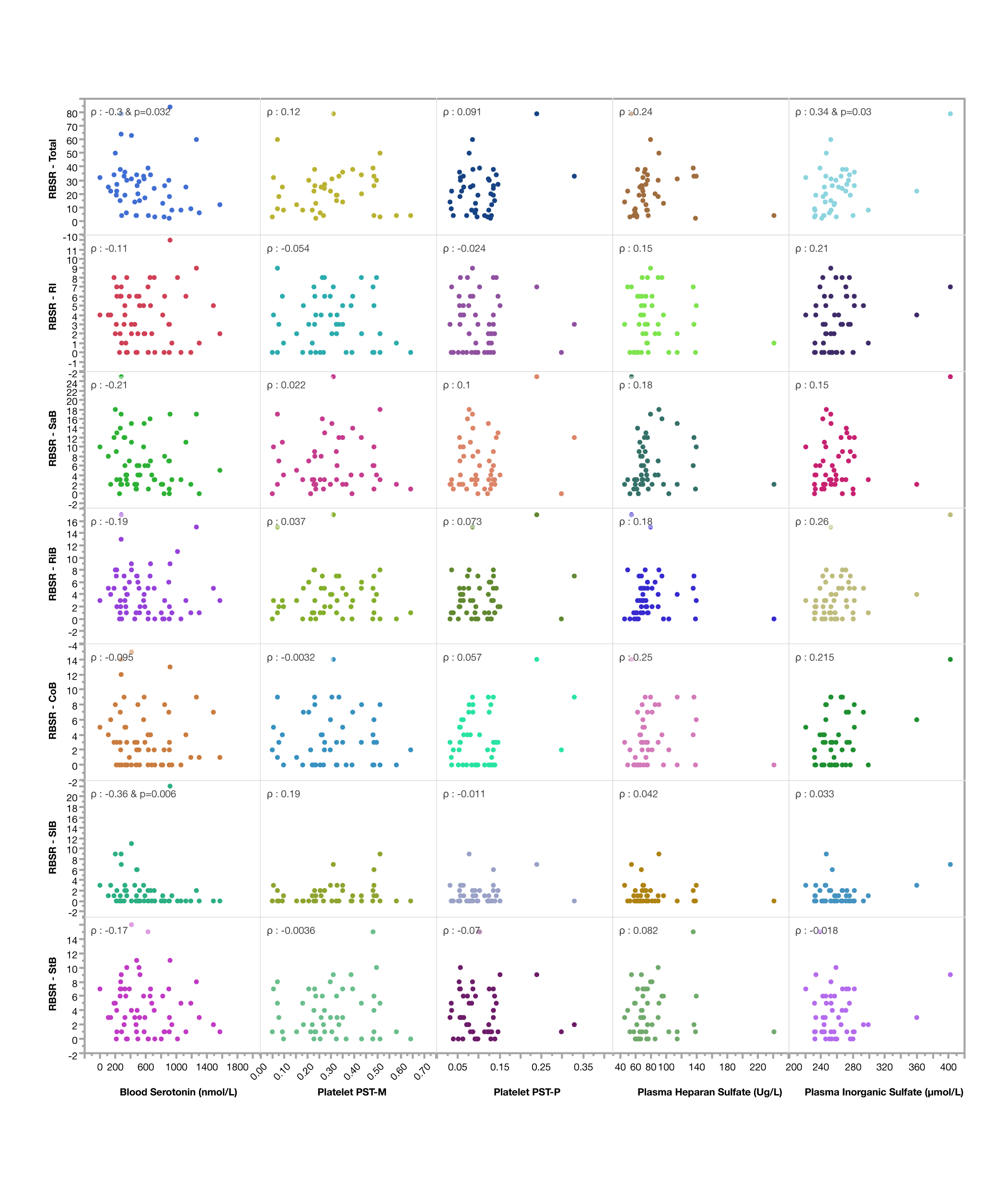
**

**Supplementary Figure 5: Correlations between subscale scores of the Repetitive Behavior Scale Revised and blood serotonin level, plasma heparan sulfate and inorganic sulfate levels, and platelet PST-P and PST-M activities in ASD individuals (n=50).** RBSR, Repetitive Behavior Scale Revised; StB, Stereotypic Behavior; SIB, Self-Injurious Behavior; CoB, Compulsive Behavior; RiB; Ritualistic Behavior; SaB, Sameness Behavior; ReB, Restricted Interests. Nominal P values <0.05 are indicated. Nominal P values are significant for the correlation between plasma inorganic sulfate and scores for the repetitive behavior scale (RBS-R), but this is driven by two patients with very high plasma inorganic sulfate and high RBS-R scores. For each association, the Spearman's rank-order correlation coefficient (ρ) is indicated as well as significant nominal p values (p<0.05). After correction for multiple testing, none of the biochemical parameters are significantly associated with the RBS-R subscale scores.

**
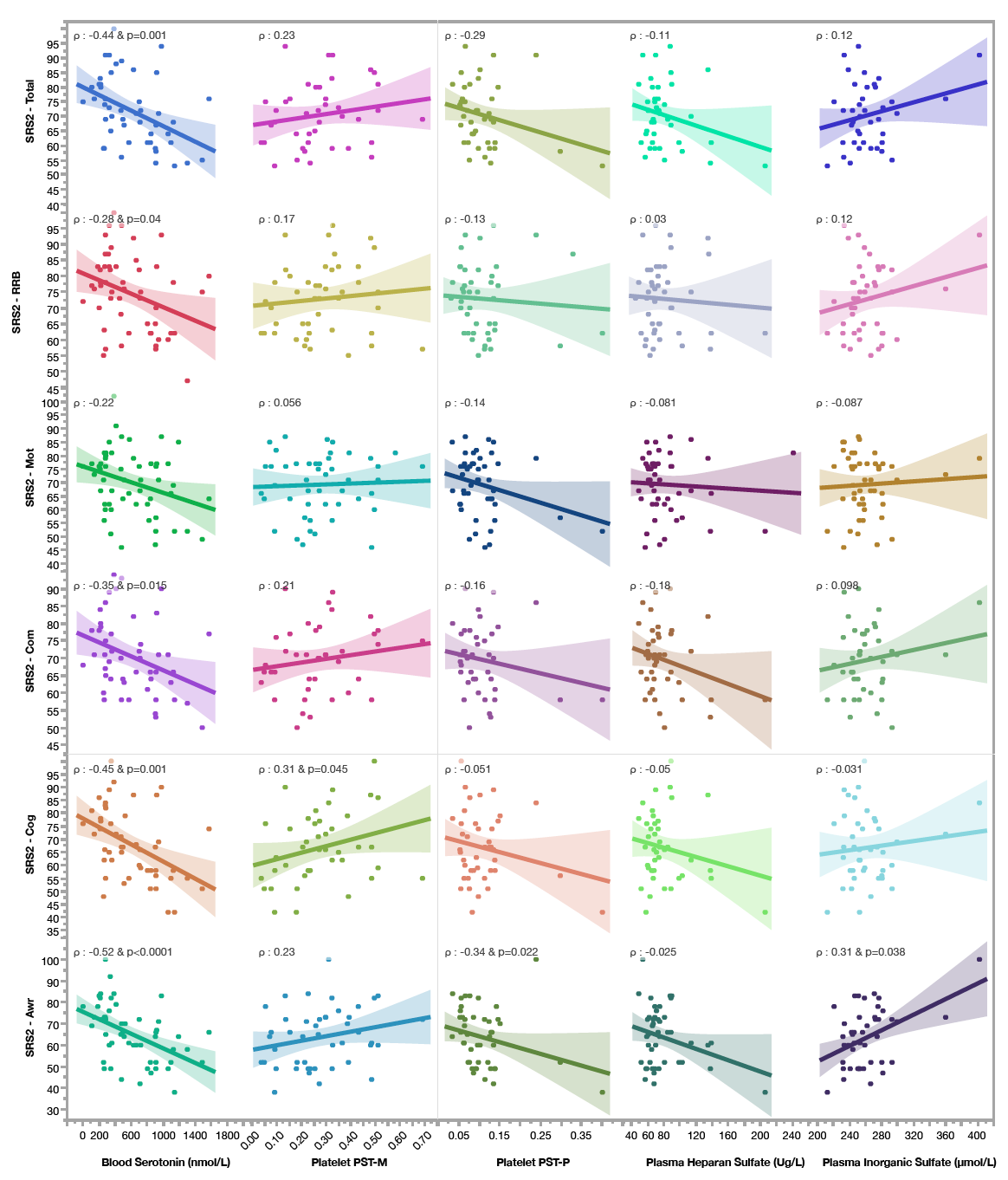
**

**Supplementary Figure 6: Correlation between subscale scores of the Social Responsiveness Scale (version 2) and blood serotonin level, plasma heparan sulfate and inorganic sulfate levels, and platelet PST-P and PST-M activities in ASD individuals (n=50).** Awr, Social Awareness; Cog, Social Cognition; Com, Social Communication; Mot, Social Motivation; RRB, Restricted Interests and Repetitive Behavior. Nominal P values <0.05 are indicated. The thresholds usually used for the SRS scores are <60 for typical individuals and respectively 60-65, 65-75 and >75 for mild, moderate and severe autistic traits.

**
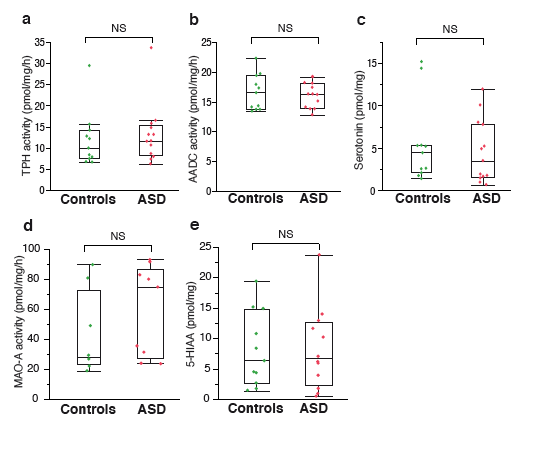
**

**Supplementary Figure 7: Serotonin synthesis and catabolism in post-mortem ilea samples from 13 patients with ASD and 11 controls.** TPH: tryptophan hydroxylase. AADC: aromatic aminoacid decarboxylase. MAO-A: monoamine oxidase, isoform A. 5-HIAA: 5-hydroxyindole acetic acid. Boxes indicate medians and quartiles. Groups were compared using Wilcoxon two-sample test.

**
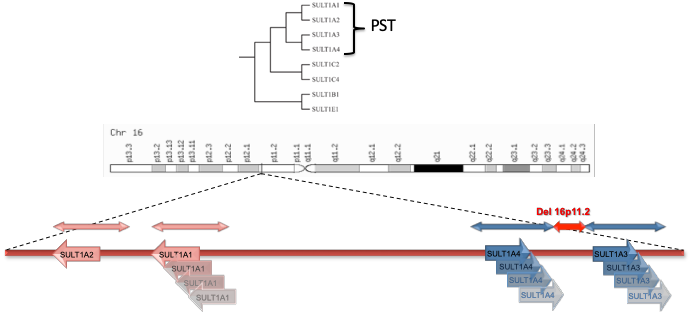
**

**Supplementary Figure 8. Phylogeny of the SULT1A/B/C gene family and location of the SULT1A1-4 genes in the human genome.** All genes are located on the chromosome 16p. The *SULT1A1*, *SULT1A3* and *SULT1A4* genes have multi-copies in the human population. The *SULT1A3* and *SUL1A4* are located within the segmental duplications flanking the 16p11.2 region associated with autism and neurodevelopmental disorders.

**Supplementary Figure 9: *SULT1A1* and *SULT1A3-4* copy number distributions in patients with ASD, their relatives and controls from all ancestries.** Quartet families include one affected child and one unaffected child.

**Supplementary Figure 10: Comparison of PST-M and PST-P activities, blood serotonin, plasma heparan sulfate and plasma inorganic sulfate levels between *SULT1A1* and *SULT1A3-4* copy numbers in patients with ASD.** Blood serotonin was measured on 245 individuals with ASD. Plasma heparan and inorganic sulfate level, and platelet PST-P and PST-M activities were assessed within 88 individuals with ASD. Biochemical traits were not normally distributed (Shapiro-Wilk test) except for PST activities. Distributions were compared using Kruskal-Wallis test for serotonin and using one-way ANOVA for PST activities. None of the biochemical parameters are significantly associated with *SULT1A1* and *SULT1A3-4* copy numbers.

**Supplementary Figure 11: Comparison of PST-M and PST-P activities, blood serotonin, plasma heparan sulfate and plasma inorganic sulfate levels between *SULT1A1* and *SULT1A3-4* copy numbers in the control group (n=88).** Biochemical traits were not normally distributed (Shapiro-Wilk test). Distributions were compared using Kruskal-Wallis test. None of the biochemical parameters are significantly associated with *SULT1A1* and *SULT1A3-4* copy numbers.

**Supplementary Table 1: Description of the populations investigated for blood biochemical parameters.**

|  |  | **Controls** | **Probands with ASD** | **Parents** | **Unaffected siblings** |
| --- | --- | --- | --- | --- | --- |
| Pagan et al, 2014 | Blood serotonin | 368 | 235 | 343 | 129 |
| **This study** | **Biochemical parameters in blood** | **106** | **97** | **138** | **56** |
|  | % females | 53% | 16% | 54% | 61% |
|  | Age (median [quartiles]) | 33[12;41] | 22[16;32] | 52[47;60] | 18[12;22] |

**Supplementary Table 2: Primers, probes and enzymes used for CNVs detection of *SULT1A1-4* genes using droplet digital PCR.**

| **Amplified region** | **Primers and probes** | **Fragment length** | **Digestion** |
| --- | --- | --- | --- |
| *SULT1A1* Exon 3 | Probe: 5' FAM-TCACCGAGCTCCCATCTTCA-MGB-NFQ 3' | 81 bp | Hae III |
|  | Forward primer: 5' CAGGGTGGTGACCTGGAG 3' |  |  |
|  | Reverse primer: 5' AATCCCTGGGGCTTTGAA 3' |  |  |
| *SULT1A2* Exon 5 | Probe: 5' FAM-CTACCACTTCTACCACATGGCCAAA-MGB-NFQ 3' | 76 bp | Alu I |
|  | Forward primer: 5' CGCAACGCAAAGGATGTG 3' |  |  |
|  | Reverse primer: 5' AGGTCCCAGGGTGAGGGTA 3' |  |  |
| *SULT1A3* Intron 6 | Probe: 5' FAM-AACCCCTGGGGCCCTAGTC-MGB-NFQ 3' | 69 bp | Alu I |
|  | Forward primer: 5’ CCCCTAGAAACCCCAAAGAG 3’ |  |  |
|  | Reverse primer: 5' CAGAGATCAATTCATTTTACTCCAGA 3' |  |  |
| *HMBS* Intron 6 | Probe: 5' VIC-CAGTGAGTTGGCAATCGAGA-MGB-NFQ 3' | 102 bp | Alu I |
|  | Forward primer: 5' GAGTGTTGACTGGTGGCAGA 3' |  |  |
|  | Reverse primer: 5' GGCACAGAATGAGGGAAGAG 3' |  |  |

Bp, base pair; FAM and VIC, fluorophores; MGB-NFQ, minor groove binder non-fluorescent quencher.

**Supplementary Table 3: Primers used for amplification and sequencing of *SULT1A1-4* genes.**

| **Amplified region** | **Primers** | **Fragment length** | **Annealing temperature** |
| --- | --- | --- | --- |
| *SULT1A1* Exons 2,3,4 | Forward primer: 5' CCCCGCCACTGAGTGGCT 3' | 758 bp | 65°C |
|  | Reverse primer: 5' CCAAAGTGCTGGGATTATGGA 3' |  |  |
| *SULT1A1* Exons 5,6 | Forward primer: 5' CCTGACTTGGCCCTACCG 3' | 656 bp | 65°C |
|  | Reverse primer: 5' CGAGAGATGAGAGCTGTGTGG 3’ |  |  |
| *SULT1A1* Exons 7,8 | Forward primer: 5' GTTGGCTCTGCAGGGTTTCT 3' | 623 bp | 60°C |
|  | Reverse primer: 5' GGCCCTCAATTCATATTTTATTCTT 3' |  |  |
| *SULT1A2* Exons 2,3,4 | Forward primer: 5' CCCCGCCACTGACTGGCC 3' | 761 bp | 65°C |
|  | Reverse primer: 5' CCTCCCAAAGTACTGAGATTGC 3' |  |  |
| *SULT1A2* Exons 5,6 | Forward primer: 5' AGCTTCCTCCTTTGCCAAA 3' | 614 bp | 60°C |
|  | Reverse primer: 5' GCAGCTGACTCAGGCACAG 3' |  |  |
| *SULT1A2* Exons 7,8 | Forward primer: 5' TGGCTCTATGGGTTTTGAAGTG 3' | 614 bp | 65°C |
|  | Reverse primer: 5' CAAATCATACTTTATTCTGGAGCCT 3' |  |  |
| *SULT1A3* Exons 1B,1C | Forward primer: 5' TCATGGGATGGAGCTGTGTA 3' | 599 bp | 58°C |
|  | Reverse primer: 5' CAGGTCTCCAGCAGGTCTGT 3' |  |  |
| *SULT1A3* Exon 1A | Forward primer: 5' CTCAACCCCACCCCTTCC 3' | 245 bp | 60°C |
|  | Reverse primer: 5' GCCTCGGCTTCTAGAATGTT 3' |  |  |
| *SULT1A3* Exons 2,3,4 | Forward primer: 5' GATTGTGCCACACTGCACTC 3' | 790 bp | 63°C |
|  | Reverse primer: 5' GTCTCCATCTCTCGCCTTCA 3' |  |  |
| *SULT1A3* Exons 5,6 | Forward primer: 5' AGGTTCCTCCTTTGCCAGTT 3' | 475 bp | 58°C |
|  | Reverse primer: 5' CTTGGTGGGTCCCTGTGA 3' |  |  |
| *SULT1A3* Exons 7,8 | Forward primer: 5' CCAGATCCCCTCTGAGGTTAG 3' | 980 bp | 58°C |
|  | Reverse primer: 5' CTGGGGGAGAGGTGACCATA 3' |  |  |

Primers used for sequencing are shown in red. Bp, base pair.

**Supplementary Table 4: *SULT1A1* and *SULT1A3-4* copy number distributions in patients with ASD, their relatives and controls from European ancestry.**

|  | ***SULT1A1* copy number** | | | | | | |  |
| --- | --- | --- | --- | --- | --- | --- | --- | --- |
|  | **% (number of subjects)** | | | | | | |  |
|  | **0** | **1** | **2** | **3** | **4** | **5** | **6** | **p ^a^** |
| **Controls** (n=95) | 0 | 5.3 (5) | 63.2 (60) | 28.4 (27) | 3.2 (3) | 0 | |  |
| **Patients** (n=341) | 0.3 (1) | 5.9 (20) | 63.9 (218) | 23.5 (80) | 5.9 (20) | 0.6 (2) | 0 | 0.82 |
| **Parents** (n=427) | 0.5 (2) | 6.3 (27) | 61.1 (261) | 28.3 (121) | 2.8 (12) | 0.7 (3) | 0.2 (1) | 1 |
| **Siblings** (n=100) | 0 | 2 (2) | 69 (69) | 21 (21) | 8 (8) | 0 | | 0.19 |
| **Affected siblings** (n=37) | 0 | 5.4 (2) | 64.9 (24) | 27.0 (10) | 2.7 (1) | 0 | | 1 |
| **Total** (n=1,000) | 0.3 (3) | 5.6 (56) | 64.1 (641) | 26.0 (260) | 4.4 (44) | 0.5 (5) | 0.1 (1) |  |
| **ASD quartet** (n=36) | 0 | 2.8 (1) | 66.7 (24) | 25.0 (9) | 5.6 (2) | 0 | | 1 |
| **Siblings quartet** (n=36) | 0 | 5.6 (2) | 66.7 (24) | 22.2 (8) | 5.6 (2) | 0 | |  |
| Caucasian American (n=362)^b^ | 0 | 4.7 (17) | 69.6 (252) | 21.0 (76) | 3.6 (13) | 1.1 (4) | | 0.54 |
| African American (n=99)^b^ | 0 | 0 | 37.4 (37) | 37.4 (37) | 21.2 (21) | 4.0 (4) | | <0.001* |
| Japanese (n=97)^c^ | 0 | 0 | 65.0 (63) | 25.8 (25) | 9.2 (9) | | | 0.04* |

Quartet families include one affected child and one unaffected child. ^a^ Distribution in the control group was compared to distributions in other groups and to the literature using Fisher’s exact test, and quartet ASD and siblings distributions were compared with each other; ^b^ Hebbring *et al.* 2007; ^c^ Yu *et al.* 2013; * P-values < 0.05.

|  | ***SULT1A3-4* copy number**  **% (number of subjects)** | | | | | |  |
| --- | --- | --- | --- | --- | --- | --- | --- |
|  | **3** | **4** | **5** | **6** | **7** | **8** | **p ^a^** |
| **Controls** (n=92) | 0 | 5.4 (7) | 38.0 (49) | 52.2 (66) | 3.3 (3) | 1.1 (2) |  |
| **Patients** (n=325) | 0 | 7.1 (23) | 32.0 (104) | 49.2 (160) | 10.5 (34) | 1.2 (4) | 0.032* |
| **Parents** (n=442) | 0.2 (1) | 5.7 (25) | 33.7 (149) | 55.4 (245) | 4.8 (21) | 0.2 (1) | 0.32 |
| **Siblings** (n=103) | 1.0 (1) | 2.9 (3) | 32.0 (33) | 55.3 (57) | 8.7 (9) | 0 | 0.09 |
| **Affected siblings** (n=32) | 0 | 6.3 (2) | 25.0 (8) | 68.8 (22) | 0 | | 0.48 |
| **Total** (n=994) | 0.2 (2) | 5.8 (58) | 33.1 (329) | 53.2 (532) | 6.7 (67) | 0.6 (6) |  |
| **ASD quartet** (n=39) | 0 | 5.1 (2) | 33.3 (13) | 61.5 (24) | 0 | | 0.65 |
| **Siblings quartet** (n=39) | 0 | 2.6 (1) | 25.6 (10) | 69.2 (27) | 2.6 (1) | 0 |  |

Quartet families include one affected child and one unaffected child. ^a^ Distribution in the control group was compared to distributions in other groups using Fisher’s exact test; * P-values < 0.05.

**Supplementary Table 5: Biochemical profiles observed in patients with ASD and controls.**

| **PST-M** | **PST-P** | **HS** | **Sulfate** | **ASD** | | **Controls** | |
| --- | --- | --- | --- | --- | --- | --- | --- |
|  |  |  |  | n | % | n | % |
| Normal | Normal | Normal | Normal | 9 | 9% | 90 | 85% |
| Normal | Normal | Normal | Low | 4 | 4% | 5 | 5% |
| Normal | Normal | Low | Normal | 4 | 4% | 5 | 5% |
| Normal | Normal | Low | Low | 0 | 0% | 0 | 0% |
| Normal | Low | Normal | Normal | 24 | 25% | 1 | 1% |
| Normal | Low | Normal | Low | 10 | 10% | 1 | 1% |
| Normal | Low | Low | Normal | 9 | 9% | 1 | 1% |
| Normal | Low | Low | Low | 5 | 5% | 0 | 0% |
| Low | Normal | Normal | Normal | 7 | 7% | 3 | 3% |
| Low | Normal | Normal | Low | 1 | 1% | 0 | 0% |
| Low | Normal | Low | Normal | 0 | 0% | 0 | 0% |
| Low | Normal | Low | Low | 1 | 1% | 0 | 0% |
| Low | Low | Normal | Normal | 10 | 10% | 0 | 0% |
| Low | Low | Normal | Low | 5 | 5% | 0 | 0% |
| Low | Low | Low | Normal | 4 | 4% | 0 | 0% |
| Low | Low | Low | Low | 4 | 4% | 0 | 0% |
|  | | | **total** | **97** | **100%** | **106** | **100%** |

References

1. Launay JM, Geoffroy C, Costa JL, Alouf JE. Purified -SH-activated toxins (streptolysin O, alveolysin): new tools for determination of platelet enzyme activities. *Thrombosis research* 1984; **33**(2)**:** 189-196.

2. Launay JM, Alouf JE. Biochemical and ultrastructural study of the disruption of blood platelets by streptolysin O. *Biochimica et biophysica acta* 1979; **556**(2)**:** 278-291.

3. Piton A *et al.* 20 ans apres: a second mutation in MAOA identified by targeted high-throughput sequencing in a family with altered behavior and cognition. *European journal of human genetics : EJHG* 2014; **22**(6)**:** 776-783.

4. Denney RM, Fritz RR, Patel NT, Abell CW. Human liver MAO-A and MAO-B separated by immunoaffinity chromatography with MAO-B-specific monoclonal antibody. *Science* 1982; **215**(4538)**:** 1400-1403.

5. Vrana SL, Dworkin SI, Vrana KE. Radioenzymatic assay for tryptophan hydroxylase: [3H]H2O release assessed by charcoal adsorption. *Journal of neuroscience methods* 1993; **48**(1-2)**:** 123-129.

6. Bowsher RR, Henry DP. Decarboxylation of p-tyrosine: a potential source of p-tyramine in mammalian tissues. *Journal of neurochemistry* 1983; **40**(4)**:** 992-1002.

7. Sterner E *et al.* Assays for determining heparan sulfate and heparin O-sulfotransferase activity and specificity. *Analytical and bioanalytical chemistry* 2014; **406**(2)**:** 525-536.
